# ECG-based identification of COPD patients at risk for atrial fibrillation and its impact on adverse clinical outcomes—a subgroup analysis of the prospective multicenter COSYCONET cohort

**DOI:** 10.1186/s12931-025-03342-2

**Published:** 2025-09-17

**Authors:** Martin Eichenlaub, Björn Christian Frye, Heiko Lehrmann, Frank Biertz, Amir Sherwan Jadidi, Klaus Kaier, Thomas Melzer, Peter Alter, Henrik Watz, Benjamin Waschki, Barbara Christine Weckler, Franziska Christina Trudzinski, Julia Dorothea Michels-Zetsche, Frederik Trinkmann, Felix Josef-Friedrich Herth, Hans-Ulrich Kauczor, Kathrin Kahnert, Rudolf Jörres, Robert Bals, Dirk Westermann, Thomas Arentz, Claus Franz Vogelmeier, Daiana Stolz, Sebastian Fähndrich, on behalf of the German COSYCONET cohort, Stefan Andreas, Jürgen Behr, Thomas Brahmer, Burkhard Bewig, Ralf Ewert, Beate Stubbe, Joachim Hans Ficker, Christian Grohé, Matthias Held, Markus Henke, Anne-Marie Kirsten, Rembert Koczulla, Juliane Kronsbein, Cornelia Kropf-Sanchen, Christian Herzmann, Michael Pfeifer, Winfried Johannes Randerath, Werner Seeger, Michael Studnicka, Christian Taube, Hartmut Timmermann, Bernd Schmeck, Tobias Welte, Hubert Wirtz

**Affiliations:** 1 Department of Pneumology, Medical Center, Faculty of Medicine, University of Freiburg, Killianstrasse 5, Freiburg, 79106 Germany; 2 Department of Cardiology and Angiology, Medical Center, Faculty of Medicine, University of Freiburg, Suedring 15, Freiburg, Bad Krozingen 79189 Germany; 3 Hannover Medical School, CAPNETZ Stiftung, Hannover, Germany; 4 Arrhythmia and Electrophysiology Section, Heart Center Lucerne, Lucerne Cantonal Hospital, Lucerne, Switzerland; 5 Institute of Medical Biometry and Statistics, Faculty of Medicine, University of Freiburg, Freiburg, Germany; 6 Department of Medicine V, LMU University Hospital, LMU Munich, Comprehensive Pneumology Center, Member of the German Center for Lung Research (DZL), Munich, Germany; 7 Department of Medicine, Pulmonary, Critical Care and Sleep Medicine, Philipps-University Marburg (UMR), German Center for Lung Research (DZL), Marburg, Germany; 8 Airway Research Center North (ARCN), Pulmonary Research Institute at LungenClinic Grosshansdorf, Grosshansdorf, Germany; 9 Pneumology, Hospital Itzehoe, Itzehoe, Germany; 10 Department of Medicine, Pulmonary and Critical Care Medicine, Clinic for Airway Infections,, University Medical Centre Marburg, Philipps-University Marburg, Marburg, Germany; 11 Department of Pneumology and Critical Care, Research (DZL), Heidelberg, Translational Lung Research Center Heidelberg (TLRC-H), Member of the German Center for Lung, Thoraxklinik Heidelberg gGmbH, Heidelberg, Germany; 12 Diagnostic and Interventional Radiology, Member of the German Center of Lung Research, University Hospital Heidelberg, Heidelberg, Germany; 13 Institute and Outpatient Clinic for Occupational, Social and Environmental Medicine, Munich, Germany; 14 Department of Internal Medicine V - Pulmonology, Allergology, Critical Care Care Medicine, Saarland University Hospital, Homburg, Germany; 15 Helmholtz Institute for Pharmaceutical Research Saarland (HIPS), Helmholtz Centre for Infection Research (HZI), Saarland University Campus, Saarbrücken, Germany

**Keywords:** Atrial fibrillation, COPD, Electrocardiography, Biomarker, Screening, MACCE

## Abstract

**Background:**

Atrial fibrillation (AF) frequently occurs in patients with chronic obstructive pulmonary disease (COPD) and is associated with adverse clinical outcomes. We aimed to identify patients at risk for AF using amplified p-wave duration (APWD) analysis on electrocardiogram (ECG) as non-invasive tool to diagnose an atrial cardiomyopathy (AtCM) which is an established risk factor for AF.

**Methods:**

This subgroup analysis of the prospective COSYCONET cohort included 2,385 COPD patients from 31 study centers with baseline sinus rhythm ECG and at least one follow-up examination. Of these, 73 patients showed AF during follow-up and were propensity-score matched to controls. APWD was measured at baseline and future major adverse cardiac and cerebrovascular events (MACCE) and health related outcome were assessed.

**Results:**

219 COPD patients (70 [64–74] years, 79.5% male) were analyzed during a follow-up of 586 (210–1137) days. APWD was significantly longer in patients with AF occurrence compared to controls (132 [125–141] ms vs. 124 [117–133] ms, *p* < 0.001) and remained significant in multivariate regression analysis (OR: 1.05 [1.01–1.09], *p* = 0.03). An APWD ≥ 131 ms was identified as best cut-off for AF prediction (62% sensitivity, 70% specificity, OR: 3.91 [2.58 to 5.95], *p* < 0.001). Patients with AF had a significantly higher MACCE rate (24.7% versus 8.2%, *p* = 0.001) and a significantly lower physical activity score (1,074 [264–4,776] vs. 2,706 [975–7,339], *p* = 0.008).

**Conclusions:**

This study demonstrates that ECG-based AtCM diagnosis identifies COPD patients at risk for AF, which was associated with a substantially elevated MACCE rate and a significantly reduced physical activity. This easy, cost-effective and widely available digital biomarker might enable early therapy initiation and prevention of adverse clinical outcomes.

**Trial registration:**

NCT01245933 on Clinical-Trials.gov (Registration date: 22.11.2010).

**Supplementary Information:**

The online version contains supplementary material available at 10.1186/s12931-025-03342-2.

## Background

Chronic obstructive pulmonary disease (COPD) is often coincident with cardiovascular diseases [1, 2]. The COSYCONET (COPD and SYstemic consequences-COmorbidities NETwork) cohort study investigates the interaction of COPD and extrapulmonal comorbidities including patients from 31 study centers [3]. Especially atrial fibrillation (AF) and heart failure frequently occur in patients with COPD and are associated with worse clinical outcomes [1, 2, 4–6]. Beyond shared common risk factors, several COPD-specific conditions (e.g. exacerbation, lung hyperinflation, and airway obstruction) are known to increase risk for AF and heart failure [4]. Some recently published studies also hypothesized that COPD-related systemic inflammation might result in an atrial cardiomyopathy (AtCM) characterized by structural and electrical remodeling [7–10]. This AtCM is linked to both electrical disturbances which favors new-onset AF and mechanical dysfunction of the atria increasing risk for heart failure [11]. Recently, we could demonstrate that atrial conduction delay caused by AtCM in patients with AF can be quantified non-invasively by measurement of the amplified p-wave duration (APWD) in digital electrocardiogram (ECG) [12, 13]. However, studies analyzing the role of AtCM in patients with COPD are lacking.

Therefore, this study aimed to analyze APWD to identify COPD patients at risk for AF and to investigate its significance for adverse clinical outcomes using data from the COSYCONET study.

## Methods

### Study population

The prospective, observational COSYCONET cohort enrolled 2,741 individuals aged ≥ 40 years with stable COPD at 31 study centers between 2010 and 2013, excluding patients with lung tumor, moderate or severe exacerbation within the last four weeks prior to inclusion, or history of major lung surgery [3]. All patients underwent assessments including digital ECG, transthoracic echocardiography, laboratory parameters, blood gas analysis, pulmonary function and tests for exercise capacity and health-related questionnaires at baseline (visit 1) and after 6, 18, 36, and 54 months (visits 2–5.

**Fig. 1:**
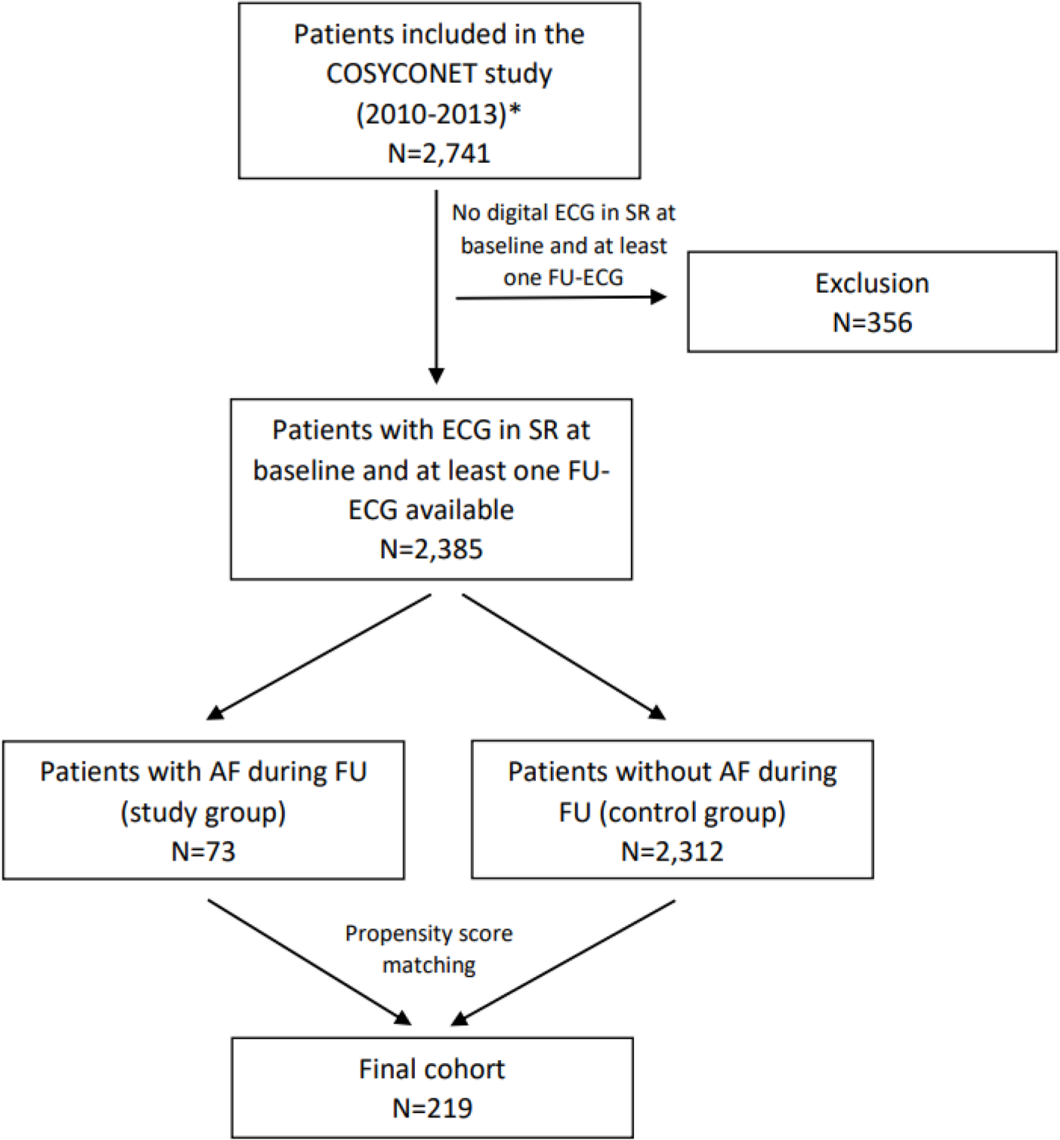
Overview of the overall cohort and the study population. AF, atrial fibrillation; ECG, electrocardiogram; SR, sinus rhythm; FU, follow-up * Individuals aged ≥ 40 years with stable COPD, excluding patients with a lung tumor, a moderate or severe exacerbation within the last four weeks prior to inclusion, or a history of major lung surgery

In our subgroup analysis, we included only patients with baseline ECG showing sinus rhythm and at least one follow-up examination with an available ECG. Of these 2,385 patients, 73 showed AF in one of the follow-up ECG-recordings and were therefore defined as study group. We conducted a propensity score matching using a 1:2 matching ratio and considering both established cardiovascular and pulmonary risk factors for AF occurrence as potential confounders to define a control group of 146 patients. An overview of the overall cohort and the study population is illustrated in Fig. 1. To exclude patients with possibly pre-existing AF diagnosis who were in sinus rhythm at baseline, we also performed a further subgroup analysis including only patients who were not receiving oral anticoagulation therapy at baseline.

The study was performed according to the declaration of Helsinki, approved by the ethical committee of the coordinating center at the University of Marburg (ID of the approval: 200/09) and all patients gave written informed consent prior to inclusion (identifier: NCT01245933 on Clinical-Trials.gov, registration date: 22.11.2010).

### Clinical and functional assessments

Details of clinical and functional assessments in COSYCONET have been described previously [3]. Lung function parameters were assessed in agreement with ATS/ERS Task Force and results expressed as % predicted using GLI Eqs. [14–17]. COPD severity (grades 1–4) and exacerbation (acute worsening, such as increased shortness of breath, increased or purulent sputum) were defined according to the GOLD criteria [18]. Hypoxemia was defined as PaO_2_ < 65 mmHg and hypercapnia as PaCO_2_ > 45 mmHg. Transthoracic echocardiography was performed with focus on diastolic and systolic dysfunction and measurement of right ventricular wall thickness to assess right ventricular remodeling as consequence of increased right ventricular afterload due to pulmonary vascular remodeling [19]. Inflammation was analyzed using a systemic inflammation panel including leucocytes, C-reactive protein (CRP), alpha 1 antitrypsin, interleukins 6 and 8, fibrinogen and tumor necrosis factor (TNF). Furthermore, we measured serum levels of high-sensitivity troponin I [20]. The BODE index was calculated using body mass index (BMI), airway obstruction, dyspnea and exercise capacity [21]. Future major adverse cardiac and cerebrovascular events (MACCE) were defined as at least one report of new-onset coronary artery disease, myocardial infarction, stroke/transient ischemic attack [TIA] and heart failure symptoms. To investigate functional parameters timed up&go test and 6 min walk distance (6MWD) were performed [22, 23]. To assess daily physical activity and quality of life we used the International Physical Activity Questionnaire (IPAQ) and the Euro Quality of life l5 dimensions questionnaire (EQ-5D), respectively and to analyze cognitive impairment the DemTect test [24, 25].

### Analysis of APWD

**Fig. 2:**
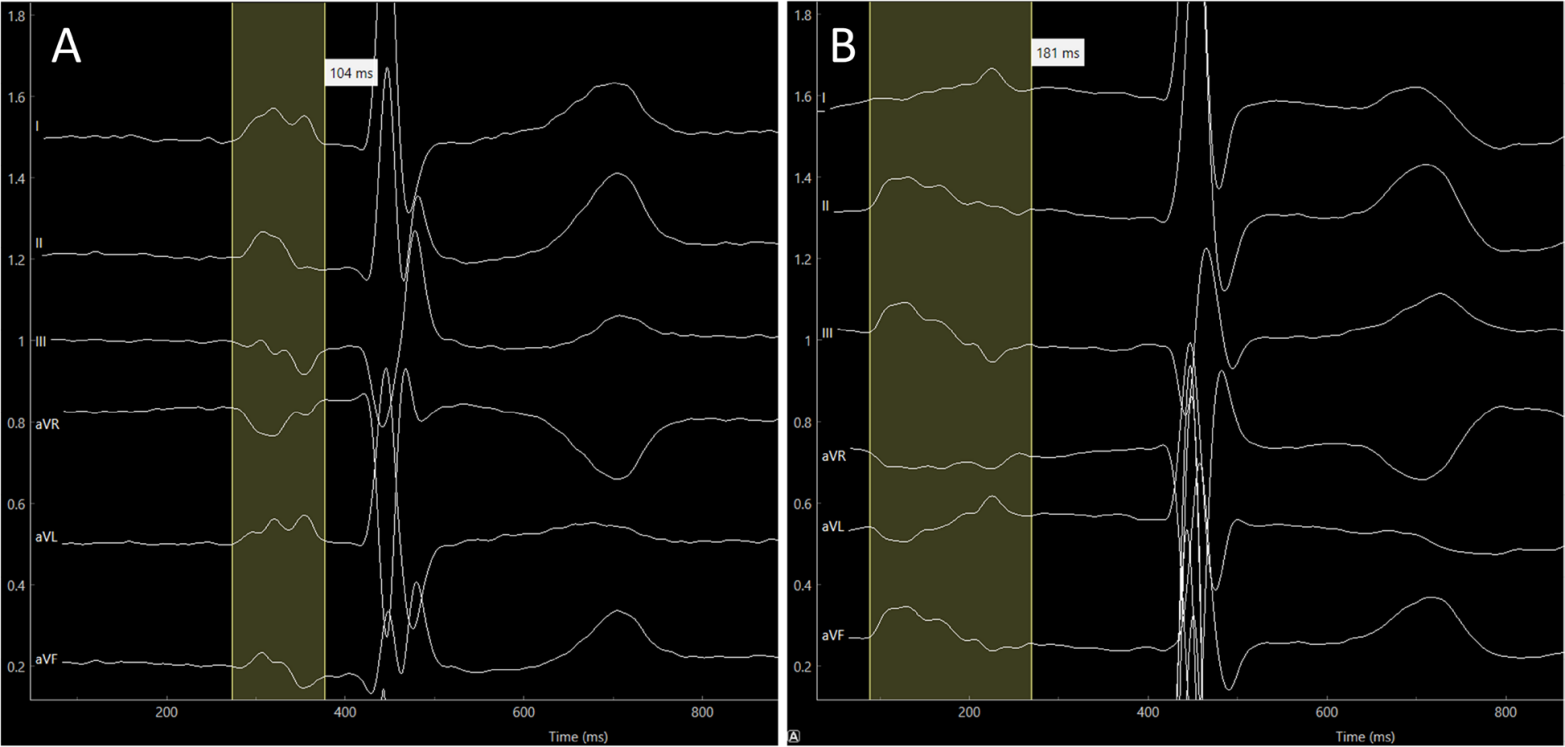
Amplified p-wave duration (APWD) measurement (only the limb leads are illustrated) between earliest onset and latest end of the p-wave in any of the ECG leads in two example patients with an APWD of 104 ms (Panel **A**) and an APWD of 181 ms (Panel **B**), respectively

Digital 12-lead ECGs were recorded using ELI 10 electrocardiograph (Mortara Instrument GmbH, Essen, Germany) in all study centers. APWD was measured in sinus rhythm ECGs at baseline of both the study and the control group between earliest onset and latest end of the p-wave in any of the 12 ECG leads by two independent cardiologists blinded to any clinical data and outcome after amplification of the ECGs to 80 mm/mV with 175 mm/s sweep speed (Fig. 2) [12, 13]. The average length of both measurements was used to calculate APWD as surrogate for AtCM.

### Endpoints

The primary endpoint was to compare APWD in COPD patients with and without occurrence of AF during follow-up. The secondary endpoints were to analyze clinical (future MACCE), functional and health related outcomes in patients with AF and to investigate the influence of hypoxemia, acute exacerbation, inflammation markers, airway obstruction and lung hyperinflation beside known cardiovascular risk factors as predictors for AtCM development.

### Statistical analysis

Statistical analysis was performed using SPSS Statistics 29 (IBM, New York, NY, USA) and Stata 18 (StataCorp, College Station, Texas, USA). A propensity score matching was conducted using the nearest neighbour method without replacement. For the matching process, the following established risk factors for AF were used as pre-defined variables: age, sex, BMI, arterial hypertension, diabetes mellitus, hyperlipidemia, obstructive sleep apnea, smoking status, pack years, long-term oxygen therapy and pulmonary function parameters for both airway obstruction (forced expiratory volume in one second [FEV_1_] and Tiffeneau-index [FEV1/forced vital capacity(FVC)]) and lung hyperinflation (residual volume/total lung capacity [RV/TLC] and transfer factor of the lung for carbon monoxide [TLCO]). Matched groups were contrasted using Mann–Whitney U test or Student´s t-test for continuous variables after testing for normal distribution using Shapiro–Wilk test or Fisher's exact test for categorical variables. Normally distributed data are given as mean ± standard deviation and skewed distributed data as median with interquartile range (1st and 3rd quartiles). Categorical data are given as number and percentage. In case of missing data, number of available data is displayed and frequency is calculated considering available data. Intraclass correlation coefficient estimates and their 95% confidence intervals (CI) on the basis of APWD measurements by two independent cardiologists were calculated on the basis of a two-way mixed-effects model for consistency. Based on the Youden method, an optimal cut-off of APWD to predict AF risk was identified performing receiver operating curve (ROC) analysis. Univariate linear regression analyses were conducted for APWD and left atrial diameter as predictors for AF risk and for clinical relevant risk factors for relevant AtCM as assessed by APWD. In the multivariate model, all variables with *P* < 0.05 in the univariate models were selected for analysis. Two-sided *p*-values are given, and statistical significance was considered as *P*-value < 0.05.

## Results

2,385 individuals included in the prospective, multicenter COSYCONET cohort were analyzed. Of these, 73 patients had a sinus rhythm ECG available at baseline, showed AF in one of the follow-up ECGs, and were therefore defined as study group. The study group was propensity score-matched to 146 controls selected from 2,312 patients with stable sinus rhythm on ECG. Baseline clinical data of both the study and the control cohort are displayed in Table 1. Median age was 70 (64–74) years with the majority of patients being male. Patients in the study cohort did not differ from the matched controls regarding age, sex, BMI, both cardiovascular and pulmonary risk factors as well as pulmonary function and laboratory parameters. Patients in the study group were significantly more often on oral anticoagulation and digitalis therapy and had a significant lower hypoxemia rate and a significant higher DemTect score.

**Table 1:** Clinical and functional characteristics at baseline (study inclusion)

	Total Cohort (*N* = 219)	Study group (*N* = 73)	Control group (*N* = 146)	*P* value
Clinical data				
Age, years	70 (64–74)	69 (64–73)	70 (64–74)	0.36
Male, N (%)	174 (79.5)	56 (76.7)	118 (80.8)	0.48
BMI, kg/m^2^	27.8 (24.8–31.5)	28 (25.2–31.0)	27.8 (24.7–31.9)	0.92
Arterial Hypertension, N (%)	141 (64.4)	45 (61.6)	96 (65.8)	0.55
Diabetes mellitus, N (%)	38 (17.5)	10 (13.7)	28 (19.2)	0.35
Hyperlipidemia, N (%)	97 (44.3)	31 (42.5)	66 (45.2)	0.77
Coronary artery disease, N (%)	63 (28.8)	21 (28.8)	42 (28.8)	1.0
History of myocardial infarction, N (%)	26 (11.9)	7 (9.6)	19 (13)	0.52
Heart failure (*N* = 201), N (%)	11 (5.5)	4 (5.9)	7 (5.3)	1.0
History of stroke/TIA, N (%)	20 (9.1)	5 (6.8)	15 (10.3)	1.0
Peripheral artery disease, N (%)	33 (15.1)	14 (19.2)	19 (13)	0.24
History of venous thrombosis, N (%)	18 (8.2)	6 (8.2)	12 (8.2)	1.0
Obstructive sleep apnea, N (%)	37 (16.9)	13 (17.8)	24 (16.4)	0.85
Smoking status:				0.24
- Current smokers, N (%)	40 (18.3)	17 (23.3)	23 (15.8)	
- Ex-smokers, N (%)	157 (71.7)	47 (64.4)	110 (75.3)	
- Never smokers, N (%)	22 (10)	9 (12.3)	13 (8.9)	
Pack years, years	41.1 (13.8–66.0)	37.8 (10.3–64.3)	41.6 (16.0–67.9)	0.31
COPD grades (*N* = 218):				0.86
- GOLD I, N (%)	37 (17)	10 (13.9)	27 (18.5)	
- GOLD II, N (%)	107 (49.1)	37 (51.4)	70 (47.9)	
- GOLD III, N (%)	62 (28.4)	21 (29.2)	41 (28.1)	
- GOLD IV, N (%)	12 (5.5)	4 (5.6)	8 (5.5)	
Therapy:				
Anticoagulation therapy, N (%)	16 (7.3)	15 (20.5)	1 (0.7)	**< 0.001**
Antiplatelet therapy, N (%)	84 (38.4)	22 (30.1)	62 (42.5)	0.08
Betablocker therapy, N (%)	57 (26)	25 (34.2)	32 (21.9)	0.07
Digitalis therapy, N (%)	8 (3.7)	6 (8.2)	2 (1.4)	**0.02**
Antiarrhythmic drug therapy:				
- Class III (Amiodarone, Dronedarone), N (%)	1 (0.5)	1 (1.4)	0 (0)	0.33
Respiratory medication:				
- LABA, N (%)	177 (80.8)	61 (83.6)	116 (79.5)	0.59
- LAMA, N (%)	144 (65.8)	47 (64.4)	97 (66.4)	0.77
- ICS, N (%)	138 (63.0)	44 (60.3)	94 (64.4)	0.56
- LABA/LAMA, N (%)	126 (57.5)	42 (57.5)	84 (57.5)	1.0
- LABA/ICS, N (%)	134 (61.2)	43 (58.9)	91 (62.3)	0.66
- LABA/LAMA/ICS, N (%)	96 (43.8)	31 (42.5)	65 (44.5)	0.89
Long-term oxygen therapy, N (%)	24 (11)	9 (12.3)	15 (10.3)	0.65
Clinical status				
Hypoxemia (*N* = 212), N (%)	98 (46.2)	26 (35.6)	72 (51.8)	**0.03**
Hypercapnia (*N* = 212), N (%)	11 (5.2)	3 (4.1)	8 (5.8)	0.75
Exacerbation within the last 12 months:				0.77
- Max. 1 and without hospital admission, N (%)	126 (57.5)	41 (56.2)	85 (58.2)	
- At least 2 or with hospital admission, N (%)	93 (42.5)	32 (43.8)	61 (41.8)	
mMRC dyspnea scale ≥ 2, N (%)	92 (42)	33 (45.2)	59 (40.4)	0.56
BODE index > 4 (*N* = 214), N (%)	24 (11.2)	8 (11.3)	16 (11.2)	1.0
DemTect score	16 (14–18)	17 (14–18)	15 (13–17)	**0.02**
EQ-5D utility score	0.89 (0.79–1.00)	0.89 (0.79–1.00)	0.89 (0.79–0.92)	0.69
6MWD, m	440 (369–498)	441 (362–494)	440 (371–498)	0.98
IPAQ score	2,772 (814–5,661)	2,930 (834–6,251)	2,466 (792–5,577)	0.86
Timed up&go test, s	6.6 (5.5–8.0)	6.6 (5.5–8.0)	6.6 (5.5–8.0)	0.91
Laboratory:				
Leukocytes, 10^9^/l	7.7 (6.3–9.2)	8.0 (6.3–9.4)	7.6 (6.2–9.1)	0.49
CRP, nmol/l	38.1 (19–66.4)	40.0 (19.0–65.7)	38.1 (16.7–66.4)	0.52
Alpha-1 antitrypsin, µmol, l	26.0 (22.8–28.9)	25.8 (22.3–28.6)	26.2 (22.8–29.2)	0.89
Interleukin 6, pg/ml	2.9 (0.6–6.9)	2.8 (0.8–6.7)	2.9 (0.4–7.7)	0.87
Interleukin 8, pg/ml	8.4 (5.3–12.2)	8.9 (5.6–13.8)	8.2 (5.1–11.5)	0.27
TNF, pg/ml	8.6 (5–14.6)	8.6 (5.4–14.6)	8.6 (4.7–14.6)	0.70
Fibrinogen, g/l	2.4 (1.8–3.3)	2.4 (1.8–3.3)	2.4 (1.8–3.3)	0.54
Troponin, ng/l	4.6 (2.6–8.1)	5.0 (2.9–8.1)	3.8 (2.6–8.3)	0.18
Creatinine, µmol/l	79.6 (70.5–90.2)	79.6 (70.7–88.4)	79.6 (69.9–92.5)	0.55
Lung function:				
FEV1 predicted, %	60 ± 20	58 ± 20	61 ± 21	0.33
FVC predicted, %	79 ± 19	77 ± 21	79 ± 17	0.39
FEV1/FVC	58 ± 13	57 ± 12	58 ± 14	0.70
sRaw_eff_, kPa s	1.49 (0.97–2.50)	1.47 (0.98–2.34)	1.51 (0.97–2.51)	0.74
ITGV, l	4.3 (3.5–5.4)	4.5 (3.6–5.5)	4.3 (3.5–5.4)	0.56
TLC, l	7.2 ± 1.5	7.2 ± 1.5	7.2 ± 1.5	0.96
RV, l	3.5 (2.9–4.4)	3.6 (2.9–4.3)	3.4 (2.9–4.5)	0.71
RV/TLC, %	52 ± 10	53 ± 11	51 ± 10	0.45
TLCO, %	60 ± 20	58 ± 18	61 ± 21	0.34
ECG findings:				
APWD, ms	127 (119–136)	132 (125–141)	124 (117–133)	**< 0010.**
Echocardiographic findings:				
LVEDD, mm	49 ± 7	50 ± 6	48 ± 7	**0.02**
LVEF, %	61 (55–69)	62 (55–70)	61 (56–69)	0.84
Relevant (at least moderate) mitral valve regurgitation (*N* = 189), N (%)	59 (31.2)	25 (39.7)	34 (27)	0.10
Left atrial diameter, mm	38 ± 7	40 ± 7	37 ± 7	**0.003**
E, m/s	0.67 (0.56–0.81)	0.73 (0.59–0.92)	0.64 (0.54–0.77)	**0.007**
A, m/s	0.78 (0.66–0.92)	0.77 (0.64–0.93)	0.78 (0.67–0.90)	0.68
E/A	0.82 (0.68–1.01)	0.89 (0.68–1.16)	0.79 (0.68–0.95)	0.06
Septal e´, cm/s	7.3 (6.0–9.0)	7.2 (6.0–9.0)	7.3 (5.9–9.0)	0.59
E/e´	8.8 (7.4–11.4)	9.1 (7.9–12.0)	8.6 (6.8–11.3)	0.23
E(dt), ms	224 (183–279)	226 (187–267)	223 (178–290)	0.81
Right ventricular wall thickness, mm	5.6 (4.3–6.9)	5.5 (4.0–7.0)	5.5 (4.3–6.7)	0.93
TAPSE, mm	24 ± 5	25 ± 5	24 ± 5	0.34

*6MWD* 6-min walk distance, *APWD* Amplified p-wave duration, *BMI* Body mass index, BODE index composed of BMI, airflow obstruction, dyspnea and 6MWD, *COPD* Chronic obstructive pulmonary disease, *CRP* C-reactive protein, E(dt) E wave deceleration time, *EQ-5D* Euro quality of life l 5 dimensions questionnaire, *FEV1* Forced expiratory volume in one second, *FVC* Forced vital capacity, *GOLD* Global initiative for chronic obstructive lung disease, *ICS* Inhaled corticosteroids, *IPAQ* International physical activity questionnaire, *ITGV* Intrathoracic gas volume, *LABA* Long-acting beta2-agonist, *LAMA* Long-acting muscarinic antagonist, *LDL* Low density lipoprotein, *LVEDD* Left ventricular end-diastolic diameter, *LVEF* Left ventricular ejection fraction, *mMRC* modified medical research council, *RV* Residual volume, *sRaw*_*eff*_, effective specific airway resistance, *TAPSE* Tricuspid annular plane systolic excursion, *TIA* Transient ischemic attack, *TLC* Total lung capacity, *TLCO* Transfer factor of the lung for carbon monoxide, *TNF* Tumor necrosis factor

An overview of the subgroup analysis including 174 patients who were not receiving oral anticoagulation therapy at baseline (58 patients in the study group and 116 propensity score-matched controls) is outlined in Table 1 in the Supplement. There was no difference in all clinical characteristics between patients in the study cohort and the matched controls.

### AtCM and risk for AF

**Fig. 3:**
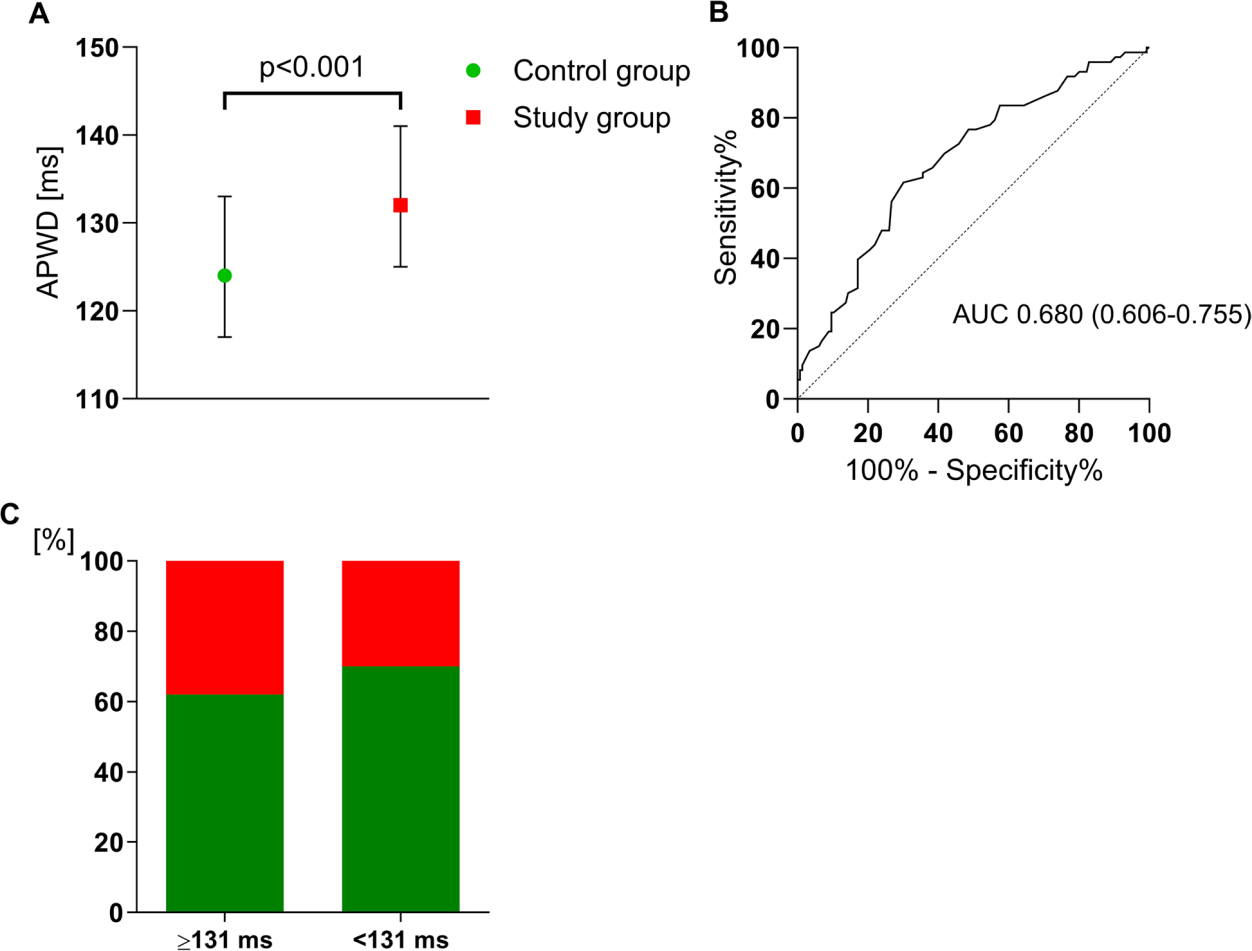
Differentiation between patients with and without atrial fibrillation (AF) during follow-up using ECG-based atrial cardiomyopathy diagnosis. Panel **A** Amplified p-wave duration (APWD) between patients with (study group) and without (control group) AF. Whiskers depict median with 25% and 75% interquartile range. Panel **B** Definition of an APWD cut-off of ≥131 ms as the best predictor for AF occurrence with an area under the curve (AUC) of 0.680. Panel **C** Accurate differentiation between patients with (sensitivity of 62%) and without (specificity of 70%) AF risk using APWD cut-off of ≥131 ms

APWD was measured by two independent investigators with an interobserver reproducibility of 0.80 (95% CI: 0.74 to 0.85). Patients in the study group had a significant prolonged APWD compared to controls (132 [125–141] ms vs. 124 [117–133] ms, *p* < 0.001, Fig. 3A). Left atrial diameter was also significantly increased in patients at risk for AF (40 ± 7 mm vs. 37 ± 7 mm, *p* = 0.003). Further, a low, but statistically significant correlation was observed between APWD and left atrial diameter (*r* = 0.27 [95% CI: 0.12 to 0.40), *p* < 0.001). In univariate regression analysis, APWD and LA diameter were both significant predictors for AF occurrence with an odds ratio of 1.05 per millisecond prolongation of APWD and 1.08 per millimeter increase for LA diameter, respectively (both *p* < 0.001). In multivariate regression analysis including both parameters only APWD remained a significant risk factor with a 5% risk increase for AF occurrence per millisecond (*p* < 0.001). ROC analysis identified an APWD ≥ 131 ms as best cut-off to predict AF occurrence with 62% sensitivity, 70% specificity and an odds ratio of 3.91 (95% CI: 2.58–5.95, *p* < 0.001, Fig. 3B + C). In the subgroup of patients with an APWD ≥ 131 ms, those who developed AF exhibited a lower prevalence of coronary artery disease, and were significantly more likely to be active smokers rather than former smokers. Furthermore, TLCO was significantly reduced in patients who developed AF within this subgroup. Detailed data for all parameters are provided in Table 2 in the Supplement.

**Table 2:** Clinical and functional characteristics at follow-up

	Total Cohort (*N* = 219)	Study group (*N* = 73)	Control group (*N* = 146)	*P* value
Clinical Outcome:				
MACCE, N (%)	30 (13.7)	18 (24.7)	12 (8.2)	**0.001**
New-onset coronary artery disease, N (%)	8 (3.7)	5 (6.8)	3 (2.1)	0.12
New-onset myocardial infarction, N (%)	3 (1.4)	2 (2.7)	1 (0.7)	0.26
New-onset heart failure (*N* = 179), N (%)	18 (10.1)	11 (18.6)	7 (5.8)	**0.02**
New-onset stroke/TIA, N (%)	4 (1.8)	2 (2.7)	2 (1.4)	0.60
Therapy:				
Anticoagulation therapy, N (%)	38 (17.4)	29 (39.7)	9 (6.2)	**< 0.001**
Antiplatelet therapy, N (%)	86 (39.3)	24 (32.9)	62 (42.5)	0.19
Betablocker therapy, N (%)	67 (30.6)	35 (47.9)	32 (21.9)	**< 0.001**
Digitalis therapy, N (%)	10 (4.6)	7 (9.6)	3 (2.1)	**0.02**
Antiarrhythmic drug therapy:				
- Class III (Amiodarone, Dronedarone), N (%)	1 (0.5)	1 (1.4)	0 (0)	0.33
Respiratory medication:				
- LABA, N (%)	165 (75.3)	59 (80.8)	106 (72.6)	0.24
- LAMA, N (%)	159 (72.6)	55 (75.3)	104 (71.2)	0.63
- ICS, N (%)	129 (58.9)	44 (60.3)	85 (58.2)	0.88
- LABA/LAMA, N (%)	135 (61.6)	48 (65.8)	87 (59.6)	0.46
- LABA/ICS, N (%)	118 (53.9)	39 (53.4)	79 (54.1)	1.0
- LABA/LAMA/ICS, N (%)	100 (45.7)	35 (47.9)	65 (44.5)	0.67
Long-term oxygen therapy, N (%)	45 (20.5)	17 (23.3)	28 (19.2)	0.48
Clinical status:				
Hypoxemia (*N* = 216), N (%)	101 (46.8)	27 (38.0)	74 (51.0)	0.08
Hypercapnia (*N* = 216), N (%)	12 (5.6)	4 (5.6)	8 (5.5)	1.0
Exacerbation within the last 12 months:				0.31
- Max. 1 and without hospital admission, N (%)	134 (61.2)	41 (56.2)	93 (63.7)	
- At least 2 or with hospital admission, N (%)	85 (38.8)	32 (43.8)	53 (36.3)	
Hospitalization since last visit, N (%)	88 (40.2)	36 (49.3)	52 (35.6)	0.06
Health status since last visit:				0.28
- Improved, N (%)	24 (11.0)	5 (6.8)	19 (13.0)	
- Idem, N (%)	105 (47.9)	34 (46.6)	71 (48.6)	
- Worse, N (%)	90 (41.1)	34 (46.6)	56 (38.4)	
mMRC dyspnea scale ≥ 2 (*N* = 218), N (%)	101 (46.3)	39 (53.4)	62 (42.8)	0.15
BODE index > 4 (*N* = 186), N (%)	24 (12.9)	11 (19.3)	13 (10.1)	0.10
DemTect score	15 (14–18)	16 (15–18)	15 (13–17)	0.09
EQ-5D utility score	0.89 (0.79–1.00)	0.89 (0.79–0.90)	0.89 (0.79–1.00)	0.27
6MWD, m	420 (358–480)	390 (310–480)	425 (366–499)	0.11
IPAQ score	2,175 (537–6,255)	1,074 (264–4,776)	2,706 (975–7,330)	**0.008**
Timed up&go test, s	7.4 (6.0–9.0)	7.6 (6.1–9.2)	7.1 (5.9–8.5)	0.24
Echocardiographic findings:				
LVEDD, mm	50 ± 7	49 ± 8	50 ± 7	0.45
LVEF, %	60 (55–66)	58 (50–65)	61 (58–68)	**0.009**
Relevant (at least moderate) mitral valve regurgitation (N = 119), N (%)	70 (58.8)	31 (68.9)	39 (52.7)	0.09
Left atrial diameter, mm	39 ± 7	42 ± 6	38 ± 7	**0.004**
E, m/s	0.68 (0.57–0.81)	0.71 (0.55–0.96)	0.68 (0.57–0.79)	0.464
A, m/s	0.80 (0.65–0.94)	0.70 (0.51–0.91)	0.82 (0.67–0.94)	**0.04**
E/A	0.84 (0.70–1.01)	1.01 (0.68–1.19)	0.80 (0.70–0.97)	0.11
Septal e´, cm/s	7.0 (5.9–8.0)	7.9 (6.0–10.2)	6.7 (5.2–7.4)	**0.01**
E/e´	10.0 (7.4–13.0)	9.7 (7.0–12.6)	10.3 (7.8–13.0)	0.43
E(dt), ms	216 (170–276)	184 (160–224)	238 (182–287)	**0.002**
Right ventricular wall thickness, mm	5.5 (4.1–7.0)	5.9 (4.4–7.1)	5.4 (4.0–7.0)	0.81
TAPSE, mm	23 ± 5	21 ± 4	24 ± 5	**0.002**

*6MWD* 6-min walk distance, BODE index composed of BMI, airflow obstruction, dyspnea and 6MWD, E (dt) E wave deceleration time, *EQ-5D* Euro quality of life l 5 dimensions questionnaire, *ICS* Inhaled corticosteroids, *IPAQ* International physical activity questionnaire, *LABA* Long-acting beta2-agonist, *LAMA* Long-acting muscarinic antagonist, *LVEDD* Left ventricular end-diastolic diameter, *LVEF* Left ventricular ejection fraction, *MACCE* Major adverse cardiac and cerebrovascular events, *mMRC* modified medical research council, *TAPSE* Tricuspid annular plane systolic excursion, *TIA* Transient ischemic attack

In the subgroup of patients without oral anticoagulation therapy at baseline, APWD was the only parameter which was significantly different between study and control group (131 [121–137] ms vs. 125 [117–134] ms, *p* = 0.008, Table 1 in the Supplement). ROC analysis identified also an APWD ≥ 131 ms as best cut-off to predict AF occurrence with 55% sensitivity, 73% specificity and an odds ratio of 3.10 (95% CI: 1.61 to 5.97, *p* < 0.001).

### Impact of AF on clinical outcome

Follow-up information of the total cohort is illustrated in Table 2. AF occurred during a median follow-up of 586 (210–1,137) days. In patients with AF, MACCE rate was significantly higher (24.7% versus 8.2%, *p* = 0.001), primarily driven by new-onset heart failure (18.6% vs. 5.8%, *p* = 0.02). Left ventricular ejection fraction (LVEF) and tricuspid annular plane systolic excursion (TAPSE) in the study group were significantly lower at follow-up (58 [50–65]% vs. 61 [58–68]%, *p* = 0.009 and 21 ± 4 vs. 24 ± 5 mm, *p* = 0.002, respectively). Furthermore, IPAQ score was significantly lower in patients in the study group compared to controls (1,074 [264–4,776] vs. 2,706 [975–7,339], *p* = 0.008). Details of the follow-up for the subgroup of patients without oral anticoagulation therapy at baseline are provided in Table 3 in the Supplement. Consistent with the findings from the total cohort, MACCE rate was significantly higher in patients with AF during follow-up (27.6% versus 6.0%, *p* < 0.001), mainly attributed to new-onset heart failure (20.0% vs. 4.3%, *p* = 0.005) and new-onset coronary artery disease (8.6% vs. 1.7%, *p* = 0.04). Furthermore, LVEF, TAPSE and IPAQ score were significantly reduced in patients with AF (58 [50–65]% vs. 65 [59–70]%, *p* = 0.003; 21 ± 3 vs. 24 ± 4 mm, *p* < 0.001; 1,707 [258–4,995] vs. 2,795 [1,188–8,435], *p* = 0.02).

**Table 3:** Uni- and multivariate regression analyses for markedly prolonged APWD

	Univariate regression analysis		Multivariate regression analysis	
	Odds ratio (95% CI)	*P* value	Odds ratio (95% CI)	*P* value
Age, years	1.04 (1.00–1.08)	**0.036**	1.05 (1.01–1.09)	**0.026**
Male	0.68 (0.34–1.35)	0.265		
BMI, kg/m^2^	1.12 (1.06–1.19)	**< 0.001**	1.13(1.06–1.20)	**< 0.001**
Arterial Hypertension	1.92 (1.07–3.45)	**0.028**	1.47 (0.79–2.75)	0.224
Diabetes mellitus	1.59 (0.79–3.20)	0.199		
Hyperlipidemia	1.32 (0.77–2.26)	0.322		
Obstructive sleep apnea	1.69 (0.83–3.44)	0.148		
Hypoxemia	1.10 (0.64–1.91)	0.727		
Exacerbation within the last 12 months	1.02 (0.59–1.75)	0.954		
Leukocytes, 10^9^/l	1.00 (0.89–1.13)	0.974		
CRP, nmol/l	1.00 (1.00–1.00)	0.225		
Alpha-1 antitrypsin, µmol, l	1.01 (0.96–1.05)	0.785		
Interleukin 6, pg/ml	1.00 (0.98–1.02)	0.687		
Interleukin 8, pg/ml	1.00 (0.96–1.05)	0.900		
TNF, pg/ml	0.99 (0.97–1.01)	0.475		
Fibrinogen, g/l	0.96 (0.76–1.21)	0.728		
FEV1, predicted %	1.00 (0.99–1.02)	0.683		
FEV1/FVC	1.01 (0.99–1.03)	0.282		
ITGV, l	0.91 (0.74–1.12)	0.373		
RV/TLC, %	0.99 (0.97–1.02)	0.574		
TLCO, %	1.00 (0.99–1.02)	0.912		
Right ventricular wall thickness, mm	1.05 (0.94–1.18)	0.405		

*BMI* Body mass index, *CRP* C-reactive protein, *FEV1* Forced expiratory volume in one second, *FVC* Forced vital capacity, *ITGV* Intrathoracic gas volume, *RV* Residual volume, *TLC* Total lung capacity, *TLCO* Transfer factor of the lung for carbon monoxide, *TNF* Tumor necrosis factor

### Risk factors for AtCM

Higher age, higher BMI and arterial hypertension were statistically significant predictors of an APWD ≥ 131 ms. In multivariate regression analysis, higher age and higher BMI remained as independent predictors for an APWD ≥ 131 ms with an odds ratio of 1.05 per year and 1.13 per kg/m^2^ increase, respectively (Table 3). The other cardiovascular risk factors as well as hypoxemia, acute exacerbation, inflammation markers, airway obstruction and lung hyperinflation were not significantly associated with an APWD ≥ 131 ms (Table 3). In the subgroup of patients without oral anticoagulation therapy at baseline higher age, higher BMI and obstructive sleep apnea were significant predictors of an APWD ≥ 131 ms in univariate regression analysis. Consistent with the findings of the total study cohort, higher age and higher BMI remained as independent predictors for an APWD ≥ 131 ms with an odds ratio of 1.05 per year and 1.12 per kg/m^2^ increase, respectively (Table 4 in the Supplement).

## Discussion

We report three main findings in this study: First, APWD as surrogate for AtCM was significantly longer in a cohort of COPD patients with AF occurrence compared to a propensity-score matched control group without AF. An APWD cut-off of ≥ 131 ms was proven suitable for identifying patients at increased risk for AF. Second, AF during follow-up was associated with both a substantially elevated MACCE rate and a significantly decreased physical activity. Third, established cardiovascular risk factors but no COPD related risk factors were significant predictors of a markedly prolonged APWD.

### Clinical relevance of AF in COPD patients

COPD significantly increases risk for AF [4, 5]. A recently published analysis by Carter et al. including 31,646 patients with COPD reported a 39% increased AF risk compared to matched controls of 158,230 patients without COPD [26]. Coincidence of AF and COPD is associated with adverse clinical outcomes [1, 2, 4, 5]. Thereby, the temporal sequence is differentially associated with prognosis, where a COPD diagnosis preceding an AF diagnosis has a higher mortality risk compared with AF diagnosis preceding COPD diagnosis [27]. In our study, we could also demonstrate that COPD patients with AF occurrence during follow-up had a statistically significant higher MACCE rate after a median follow-up of 586 (210–1,137) days compared to propensity score-matched controls without AF (24.7% vs. 8.2%, *p* = 0.001). Furthermore, physical activity was significantly reduced in patients with AF (IPAQ score: 1,074 [264–4,776] vs. 2,706 [975–7,339], *p* = 0.008, respectively) underlining the impact of AF on patient-centered outcome as physical activity is an important feature of daily life [28]. These results were confirmed after excluding patients with possibly preexisting AF diagnosis and oral anticoagulation therapy at baseline, with AF patients showing a higher MACCE rate (27.6% vs. 6.0%), a reduced LVEF (58% vs. 65%), an impaired TAPSE (21 vs. 24 mm) and a reduced IPAQ score (1,707 vs. 2,795), respectively (all *p* < 0.05).

### Risk factors for AF in COPD patients

Multiple factors contribute to the development of AF in COPD patients [4, 29]. Beyond shared common risk factors such as higher age, smoking and chronic inflammation, COPD-specific conditions like exacerbations, lung hyperinflation, hypoxemia, and hypercapnia causing pulmonary vascular constriction, airway obstruction with lower FEV1, and emphysema increase AF risk [4]. Additionally, certain COPD medications (especially short-acting beta2-agonists and oral corticosteroids) and comorbidities such as sleep apnea, arterial hypertension and obesity have been associated with an increased AF risk [4, 30, 31]. However, the precise pathophysiological mechanisms between COPD and AF remain unclear. This might be the reason that, to date, no prediction model for AF in patients with COPD has been established. Therefore, in order to find a more specific and novel risk marker we conducted a propensity score matching including most of the proposed established risk factors for AF development. We could demonstrate that AtCM is an important risk factor for AF in patients with COPD. A prolonged APWD as surrogate for an AtCM was the only significant risk factor for AF in our cohort in multivariate regression analysis, underscoring its potential as a novel diagnostic risk marker in COPD patients.

### Role of AtCM in COPD patients

The current EHRA/HRS/APHRS/LAHRS consensus statement defines an AtCM as “any complex of structural, architectural, contractile or electrophysiological changes affecting the atria with the potential to produce clinically relevant manifestations” [32]. The atrial changes are associated with an atrial dilatation, contractile dysfunction of the left atrium and interatrial conduction disturbances [32]. Left atrial dilatation can be analyzed in transthoracic echocardiography which can, however, only be performed by a cardiologist restricting the overall application [19, 32]. An easier, more cost-effective and widely available tool is therefore quantification of the interatrial conduction disturbances in ECG [32]. Our group recently demonstrated that measurement of the APWD on digital ECG is able to identify patients with relevant AtCM [12, 13]. In COPD patients, AtCM might be caused by systematic inflammation in particular during acute exacerbation, hypoxemia, airway obstruction and hyperinflation triggering pulmonary vascular constriction [4]. Hiram et al. demonstrated that induced right heart disease in Wistar rats resulted in significant fibrosis in both the right and left atria, which produced significantly increased conduction abnormalities and a significantly increased AF vulnerability [33]. To the best of our knowledge, the current study is the first investigating if ECG-based AtCM diagnosis is able to predict AF risk in COPD patients. We could demonstrate that patients with AF occurrence had a statistically significant prolonged APWD compared to matched controls (132 [125–141)] ms vs. 124 [117–133] ms, *p* < 0.001). This was also apparent in the subgroup of patients without oral anticoagulation therapy at baseline. Left atrial diameter as another surrogate for an AtCM was also significantly different between patients with and without AF (40 ± 7 mm vs. 37 ± 7 mm, *p* = 0.003). However, in multivariate regression analysis including both parameters only APWD remained a significant risk factor with a 5% risk increase for AF occurrence per millisecond prolongation (*p* < 0.001). We also defined an APWD cut-off of ≥ 131 ms to predict AF occurrence. This threshold is similar to the recently published APWD cut-off of ≥ 136 ms which was reported as optimal threshold to identify patients with AF diagnosis based on the analysis of their ECGs recorded in sinus rhythm [34]. We could identify higher age, higher BMI, obstructive sleep apnea and arterial hypertension as predictors for a markedly prolonged APWD which is in line with previous studies [32, 35]. In contrast COPD-related risk factors such as inflammation, exacerbation, hypoxemia, airway obstruction, hyperinflation and right ventricular remodeling were not associated with AtCM. This might be explained by the fact that most of these factors were included in the matching process in order to find a novel, more specific risk factor for AF in COPD patients. Interestingly, within the subgroup of patients with markedly prolonged APWD, those who developed AF exhibited a significantly lower prevalence of coronary artery disease, a higher rate of active smoking, and a reduced TLCO compared to patients who did not develop AF. Thus, in COPD patients with ECG-based evidence of AtCM, respiratory factors (such as active smoking and reduced TLCO) appear to play an additional role in the development of AF [4].

### Future perspectives

This study emphasizes the role of AtCM in AF pathogenesis among COPD patients. Thereby, especially cardiovascular risk factors such as higher BMI, arterial hypertension and obstructive sleep apnea were associated with AtCM highlighting the need of an interdisciplinary collaboration between pulmonologists and cardiologists to optimize risk factors from both disciplines. This is in accordance with the recently published European Society of Cardiology guidelines on AF, which underscore the importance of managing cardiovascular risk factors as crucial part of the therapeutic regimen for AF [36]. Further large-scale randomized studies are needed to investigate if COPD patients with relevant AtCM will benefit from more extensive AF screening and a more strict adjustment of cardiovascular risk factors to prevent future AF which significantly increases risk for MACCE.

### Limitations

First, we defined a novel threshold for APWD to diagnose relevant AtCM in this proof-of-concept study without external validation. Future large-scale studies are required to confirm this cut-off. Second, we analyzed a selected subgroup from the COSYCONET cohort which accounted for the higher proportion of male patients compared to the overall COSYCONET cohort. Third, patients in the study cohort were significantly more often on oral anticoagulation therapy at baseline suggesting that AF may have been a pre-existing diagnosis in a majority of these patients before study inclusion. To address this, we conducted a subgroup analysis of patients without anticoagulation therapy at baseline, which corroborated the findings from the total cohort. Fourth, AF screening was only performed during standardized follow-up visits using 12-lead ECGs. Therefore, precise time to event is missing and AF rate might be underestimated due to lack of a systematic Holter-ECG screening. Fifth, data on right heart parameters were limited. Especially, right heart catheterization to evaluate impact of pulmonary hypertension has not been performed and systolic pulmonary arterial pressure was not routinely measured. Instead, we used right ventricular wall thickness as surrogate for right ventricular remodeling. Sixth, physical activity was assessed solely through questionnaires, without the use of objective measures such as pedometers or accelerometers. Seventh, systematic measurements of natriuretic peptides, including ANP and BNP as recognized biomarkers associated with AF risk, were not available in our study. However, a notable limitation of these biomarkers is their lack of specificity, as elevated levels are observed in a variety of cardiac conditions. In contrast, APWD is a more specific marker for AtCM. Eighth, although a COPD cohort has been examined, it cannot be deduced that the findings are specific for COPD. Also, other risk factors for AF, as commonly occurring in a population exhibiting the present characteristics, could be of relevance.

## Conclusions

ECG-based diagnosis of AtCM in patients with COPD allows identification of patients at risk for AF which was associated with a substantially elevated MACCE rate and a significantly reduced physical activity. COPD patients with AtCM should therefore be monitored more closely and receive interdisciplinary follow-up by both pulmonologists and cardiologists to optimize risk factors.

## Supplementary Information

Supplementary Material 1

## Data Availability

The datasets analysed during the current study are available from the corresponding author on reasonable request.

## Abbreviations

6MWD: 6 Minutes walk distance
AtCM: Atrial cardiomyopathy
AF: Atrial fibrillation
APWD: Amplified p-wave duration
BMI: Body mass index
CI: Confidence interval
COPD: Chronic obstructive pulmonary disease
CRP: C-reactive protein
ECG: Electrocardiogram
EQ-5D: Euro Quality of life l 5 dimensions questionnaire
FEV_1_: Forced expiratory volume in one second
FVC: Forced vital capacity
GOLD: Global Initiative for Chronic Obstructive Lung Disease
IPAQ: International Physical Activity Questionnaire
LVEF: Left ventricular ejection fraction
MACCE: Major adverse cardiac and cerebrovascular events
ROC: Receiver operating curve
RV: Residual volume
TAPSE: Tricuspid annular plane systolic excursion
TIA: Transient ischemic attack
TNF: Tumor necrosis factor
TLC: Total lung capacity
TLCO: Transfer factor of the lung for carbon monoxide
