# Supplementary material for: ECG-based identification of COPD patients at risk for atrial fibrillation and its impact on adverse clinical outcomes—a subgroup analysis of the prospective multicenter COSYCONET cohort
Source: Respir Res. 2025 Sep 17;26:272. doi: 10.1186/s12931-025-03342-2 (PMC12442265; doi:10.1186/s12931-025-03342-2)
Supplement: Supplementary file 1 — Supplementary Material 1 [file 12931_2025_3342_MOESM1_ESM.docx]

**Supplemental Material**

e-Table 1. Clinical and functional characteristics at baseline (study inclusion) of the subgroup of patients without oral anticoagulation therapy.

|  | Total Cohort (N=174) | Study group (N=58) | Control group (N=116) | *P* value |
| --- | --- | --- | --- | --- |
| **Clinical data** | | | | |
| Age, years | 70 (64-74) | 69 (63-72) | 70 (64-74) | 0.21 |
| Male, N (%) | 141 (81.0) | 46 (79.3) | 95 (81.9) | 0.48 |
| BMI, kg/m^2^ | 28.0 (25.1-31.6) | 27.9 (25.1-30.2) | 28.2 (25.1-32.0) | 0.70 |
| Arterial Hypertension, N (%) | 107 (61.5) | 35 (60.3) | 72 (62.1) | 0.87 |
| Diabetes mellitus, N (%) | 29 (16.7) | 6 (10.3) | 23 (19.8) | 0.13 |
| Hyperlipidemia, N (%) | 77 (44.3) | 25 (43.1) | 52 (44.8) | 0.87 |
| Coronary artery disease, N (%) | 50 (28.7) | 16 (27.6) | 34 (29.3) | 0.86 |
| History of myocardial infarction, N (%) | 23 (13.2) | 6 (10.3) | 17 (14.7) | 0.49 |
| Heart failure (N=159), N (%) | 8 (5.0) | 1 (1.9) | 7 (6.7) | 0.27 |
| History of stroke/TIA, N (%) | 18 (10.3) | 5 (8.6) | 13 (11.2) | 0.79 |
| Peripheral artery disease, N (%) | 25 (14.4) | 9 (15.5) | 16 (13.8) | 0.82 |
| History of venous thrombosis, N (%) | 14 (8.0) | 4 (6.9) | 10 (8.6) | 0.78 |
| Obstructive sleep apnea, N (%) | 34 (19.5) | 13 (22.4) | 21 (18.1) | 0.55 |
| Smoking status:   - Current smokers, N (%) - Ex-smokers, N (%) - Never smokers, N (%) | 30 (17.2)  124 (71.3)  20 (11.5) | 12 (20.7)  38 (65.5)  8 (13.8) | 18 (15.5)  86 (74.1)  12 (10.3) | 0.50 |
| Pack years, years | 39.8 (13.7-65.5) | 34.0 (10.4-63.5) | 41.6 (16.5-66.0) | 0.34 |
| COPD grades (N=173):   - GOLD I, N (%) - GOLD II, N (%) - GOLD III, N (%) - GOLD IV, N (%) | 30 (17.3)  83 (48.0)  49 (28.3)  11 (6.4) | 7 (12.3)  28 (49.1)  19 (33.3)  3 (5.3) | 23 (19.8)  55 (47.4)  30 (25.9)  8 (6.9) | 0.53 |
| **Therapy** | | | | |
| Antiplatelet therapy, N (%) | 70 (40.2) | 19 (32.8) | 51 (44.0) | 0.19 |
| Betablocker therapy, N (%) | 45 (25.9) | 16 (27.6) | 29 (25.0) | 0.72 |
| Digitalis therapy, N (%) | 3 (1.7) | 2 (3.4) | 1 (0.9) | 0.26 |
| Respiratory medication:   - LABA, N (%) - LAMA, N (%) - ICS, N (%) - LABA/LAMA, N (%) - LABA/ICS, N (%) - LABA/LAMA/ICS, N (%) | 143 (82.2)  111 (63.8)  111 (63.8)  97 (55.7)  109 (62.6)  75 (43.1) | 49 (84.5)  35 (60.3)  38 (65.5)  31 (53.4)  37 (63.8)  26 (44.8) | 94 (81.0)  76 (65.5)  73 (62.9)  66 (59.6)  72 (62.1)  49 (42.2) | 0.68  0.51  0.87  0.75  0.87  0.75 |
| Long-term oxygen therapy, N (%) | 18 (10.3) | 6 (10.3) | 12 (10.3) | 1.0 |
| **Clinical status** | | | | |
| Hypoxemia (N=169), N (%) | 79 (46.7) | 22 (37.9) | 57 (51.4) | 0.11 |
| Hypercapnia (N=169), N (%) | 9 (5.3) | 2 (3.4) | 7 (6.3) | 0.72 |
| Exacerbation within the last 12 months:   - Max. 1 and without hospital admission, N (%) - At least 2 or with hospital admission, N (%) | 97 (55.7)  77 (44.3) | 31 (53.4)  27 (46.6) | 66 (56.9)  50 (43.1) | 0.75 |
| mMRC dyspnea scale ≥2, N (%) | 74 (42.5) | 26 (44.8) | 48 (41.4) | 0.75 |
| BODE index >4 (N=169), N (%) | 17 (10.1) | 5 (8.9) | 12 (10.6) | 1.0 |
| DemTect score | 15 (14-18) | 17 (14-18) | 15 (13-18) | 0.05 |
| EQ-5D utility score | 0.89 (0.79-1.00) | 0.89 (0.79-1.00) | 0.89 (0.79-0.90) | 0.51 |
| 6MWD, m | 440 (369-494) | 437 (369-502) | 440 (368-495) | 0.62 |
| IPAQ score | 2,296 (722-6,251) | 2,478 (815-6,518) | 2,106 (693-5,706) | 0.85 |
| Timed up&go test, s | 6.7 (5.5-8.0) | 6.5 (5.4-8.0) | 6.8 (5.6-8.1) | 0.35 |
| **Laboratory** | | | | |
| Leukocytes, 10^9^/l | 7.7 (6.2-9.3) | 8.0 (6.2-9.5) | 7.6 (6.2-9.1) | 0.59 |
| CRP, nmol/l | 41.9 (19.0-67.8) | 42.5 (19.0-63.8) | 40.0 (19.0-76.3) | 0.91 |
| Alpha-1 antitrypsin, µmol, l | 26.1 (22.7-28.5) | 25.6 (22.3-28.2) | 26.7 (22.8-29.2) | 0.44 |
| Interleukin 6, pg/ml | 2.9 (0.8-8.5) | 2.8 (1.0-9.0) | 2.9 (0.4-7.7) | 0.62 |
| Interleukin 8, pg/ml | 8.5 (5.7-12.4) | 9.0 (6.1-14.5) | 8.2 (5.3-11.9) | 0.21 |
| TNF, pg/ml | 8.6 (5.0-14.4) | 10.2 (5.5-16.5) | 8.0 (4.6-13.5) | 0.18 |
| Fibrinogen, g/l | 2.4 (1.8-3.2) | 2.4 (1.8-3.1) | 2.4 (1.8-3.2) | 0.62 |
| Troponin, ng/l | 4.7 (2.7-8.6) | 4.9 (2.7-8.0) | 4.6 (2.6-8.6) | 0.59 |
| Creatinine, µmol/l | 79.6 (70.7-90.2) | 74.3 (70.5-87.1) | 79.6 (70.7-94.0) | 0.16 |
| **Lung function** | | | | |
| FEV1 predicted, % | 60±21 | 57±20 | 61±21 | 0.48 |
| FVC predicted, % | 78±19 | 76±22 | 80±17 | 0.78 |
| FEV1/FVC | 58±14 | 57±11 | 58±14 | 0.55 |
| sRaw_eff_, kPa s | 1.49 (0.96-2.57) | 1.46 (0.92-2.38) | 1.54 (0.97-2.62) | 0.43 |
| ITGV, l | 4.3 (3.5-5.5) | 4.5 (3.6-5.5) | 4.2 (3.4-5.4) | 0.55 |
| TLC, l | 7.2±1.5 | 7.2±1.6 | 7.2±1.5 | 0.89 |
| RV, l | 3.5 (2.9-4.4) | 3.6 (2.9-4.3) | 3.4 (2.9-4.6) | 0.78 |
| RV/TLC, % | 52±11 | 53±11 | 52±10 | 0.59 |
| TLCO, % | 61±20 | 59±18 | 62±21 | 0.51 |
| **ECG findings** | | | | |
| APWD, ms | 126 (118-135) | 131 (121-137) | 125 (117-134) | **0.008** |
| **Echocardiographic findings** | | | | |
| LVEDD, mm | 49±7 | 50±8 | 48±7 | 0.18 |
| LVEF, % | 62 (55-69) | 65 (55-70) | 61 (56-68) | 0.30 |
| Relevant (at least moderate) mitral valve regurgitation (N=153), N (%) | 45 (29.4) | 17 (33.3) | 28 (27.5) | 0.46 |
| Left atrial diameter, mm | 38±7 | 40±7 | 37±7 | 0.06 |
| E, m/s | 0.67 (0.54-0.80) | 0.73 (0.58-0.88) | 0.64 (0.54-0.77) | 0.07 |
| A, m/s | 0.78 (0.65-0.93) | 0.78 (0.65-0.93) | 0.78 (0.66-0.90) | 0.89 |
| E/A | 0.79 (0.67-1.01) | 0.85 (0.66-1.10) | 0.78 (0.68-0.98) | 0.31 |
| Septal e´, cm/s | 7.3 (6.0-9.0) | 7.4 (6.0-9.0) | 7.2 (6.0-9.0) | 0.58 |
| E/e´ | 8.8 (7.4-11.3) | 9.1 (7.9-11.2) | 8.8 (7.0-11.3) | 0.50 |
| E(dt), ms | 223 (183-289) | 222 (187-266) | 224 (180-292) | 0.99 |
| Right ventricular wall thickness, mm | 5.5 (4.1-7.0) | 5.6 (4.0-7.0) | 5.2 (4.2-6.6) | 0.57 |
| TAPSE, mm | 24±5 | 25±5 | 24±5 | 0.21 |

6MWD, 6-min walk distance; APWD, amplified p-wave duration; BMI, body mass index; BODE index composed of BMI, airflow obstruction, dyspnea and 6MWD; COPD, chronic obstructive pulmonary disease; CRP, C-reactive protein; E(dt) E wave deceleration time; EQ-5D, euro quality of life l 5 dimensions questionnaire; FEV1, forced expiratory volume in one second; FVC, forced vital capacity; GOLD, global initiative for chronic obstructive lung disease; ICS, inhaled corticosteroids; IPAQ, international physical activity questionnaire; ITGV, intrathoracic gas volume; LABA, long-acting beta2-agonist; LAMA, long-acting muscarinic antagonist; LDL, low density lipoprotein; LVEDD, left ventricular end-diastolic diameter; LVEF, left ventricular ejection fraction; mMRC, modified medical research council; RV, residual volume; sRaw_eff_, effective specific airway resistance; TAPSE, tricuspid annular plane systolic excursion; TIA, transient ischemic attack; TLC, total lung capacity; TLCO, transfer factor of the lung for carbon monoxide; TNF, tumor necrosis factor

e-Table 2. Clinical and functional characteristics at baseline (study inclusion) of the subgroup of patients with APWD ≥131 ms.

|  | Total cohort (N=86) | Study group (N=45) | Control group (N=41) | *P* value |
| --- | --- | --- | --- | --- |
| **Clinical data** | | | | |
| Age, years | 70 (66-73) | 70 (67-73) | 71 (66-75) | 0.28 |
| Male, N (%) | 71 (82.6) | 36 (80) | 35 (85.4) | 0.58 |
| BMI, kg/m^2^ | 30.3±5.0 | 29.4±4.8 | 31.4±5.0 | 0.06 |
| Arterial Hypertension, N (%) | 63 (73.3) | 32 (71.1) | 31 (75.6) | 0.81 |
| Diabetes mellitus, N (%) | 19 (22.1) | 7 (15.6) | 12 (29.3) | 0.19 |
| Hyperlipidemia, N (%) | 43 (50.0) | 21 (46.7) | 22 (53.7) | 0.67 |
| Coronary artery disease, N (%) | 30 (34.9) | 11 (24.4) | 19 (46.3) | **0.04** |
| History of myocardial infarction, N (%) | 9 (10.5) | 3 (6.7) | 6 (14.6) | 0.30 |
| Heart failure (N=80), N (%) | 5 (6.3) | 3 (7.1) | 2 (5.3) | 1.0 |
| History of stroke/TIA, N (%) | 6 (7.0) | 2 (4.4) | 4 (9.8) | 0.42 |
| Peripheral artery disease, N (%) | 18 (20.9) | 10 (22.2) | 8 (19.5) | 0.80 |
| History of venous thrombosis, N (%) | 8 (9.3) | 4 (8.9) | 4 (9.8) | 1.0 |
| Obstructive sleep apnea, N (%) | 19 (22.1) | 9 (20.0) | 10 (24.4) | 0.80 |
| Smoking status:   - Current smokers, N (%) - Ex-smokers, N (%) - Never smokers, N (%) | 13 (15.1)  63 (73.3)  10 (11.6) | 11 (24.4)  29 (64.4)  5 (11.1) | 2 (4.9)  34 (82.9)  5 (12.2) | **0.04** |
| Pack years, years | 43.0 (9.4-65.3) | 43.0 (9.9-66.1) | 40.5 (7.9-65.5) | 0.71 |
| COPD grades (N=85):   - GOLD I, N (%) - GOLD II, N (%) - GOLD III, N (%) - GOLD IV, N (%) | 17 (19.8)  42 (48.8)  22 (25.6)  4 (4.7) | 6 (13.6)  24 (54.5)  11 (25.0)  3 (6.8) | 11 (26.8)  18 (43.9)  11 (26.8)  1 (2.4) | 0.36 |
| **Therapy** | | | | |
| Anticoagulation therapy, N (%) | 13 (15.1) | 13 (28.9) | 0 (0) | **<0.001** |
| Antiplatelet therapy, N (%) | 39 (45.3) | 14 (31.1) | 25 (61.0) | **0.009** |
| Betablocker therapy, N (%) | 37 (43.0) | 20 (44.4) | 17 (41.5) | 0.83 |
| Digitalis therapy, N (%) | 4 (4.7) | 4 (8.9) | 0 (0) | 0.12 |
| Antiarrhythmic drug therapy:   - Class III (Amiodarone, Dronedarone), N (%) | 1 (1.2) | 1 (2.2) | 0 (0) | 1.0 |
| Respiratory medication:   - LABA, N (%) - LAMA, N (%) - ICS, N (%) - LABA/LAMA, N (%) - LABA/ICS, N (%) - LABA/LAMA/ICS, N (%) | 67 (77.9)  52 (60.5)  51 (59.3)  45 (52.3)  49 (57.0)  31 (36.0) | 39 (86.7)  29 (64.4)  27 (60.0)  26 (57.8)  27 (60.0)  17 (37.8) | 28 (68.3)  23 (56.1)  24 (58.5)  19 (46.3)  22 (53.7)  14 (34.1) | 0.07  0.51  1.0  0.39  0.66  0.82 |
| Long-term oxygen therapy, N (%) | 11 (12.8) | 7 (15.6) | 4 (9.8) | 0.53 |
| **Clinical status** | | | | |
| Hypoxemia (N=84), N (%) | 40 (47.6) | 18 (40.0) | 22 (56.4) | 0.19 |
| Hypercapnia (N=84), N (%) | 4 (4.8) | 2 (4.4) | 2 (5.1) | 1.0 |
| Exacerbation within the last 12 months:   - Max. 1 and without hospital admission, N (%) - At least 2 or with hospital admission, N (%) | 50 (58.1)  36 (41.9) | 26 (57.8)  19 (42.2) | 24 (58.5)  17 (41.5) | 1.0 |
| mMRC dyspnea scale ≥2, N (%) | 36 (41.9) | 23 (51.1) | 13 (31.7) | 0.08 |
| BODE index >4 (N=85), N (%) | 11 (12.9) | 6 (13.6) | 5 (12.2) | 1.0 |
| DemTect score | 17 (14-18) | 17 (15-18) | 16 (14-18) | 0.11 |
| EQ-5D utility score | 0.89 (0.79-1.00) | 0.89 (0.79-1.00) | 0.89 (0.79-0.95) | 0.97 |
| 6MWD, m | 440 (371-499) | 430 (311-487) | 443 (376-508) | 0.45 |
| IPAQ score | 2,826 (849-6,345) | 3,066 (876-6,318) | 2,772 (647-6,879) | 0.94 |
| Timed up&go test, s | 7.0 (5.6-8.0) | 6.6 (5.5-8.0) | 7.0 (6.0-8.1) | 0.50 |
| **Laboratory** | | | | |
| Leukocytes, 10^9^/l | 7.6 (6.2-9.1) | 7.6 (6.3-9.2) | 7.6 (6.0-9.1) | 0.93 |
| CRP, nmol/l | 35.2 (18.1-59.5) | 34.3 (19.1-59.5) | 36.4 (11.8-62.9) | 0.55 |
| Alpha-1 antitrypsin, µmol, l | 25.9 (22.8-28.9) | 25.9 (22.7-29.7) | 25.9 (22.7-28.4) | 0.53 |
| Interleukin 6, pg/ml | 3.0 (0.6-6.7) | 3.2 (0.8-6.7) | 2.3 (0.3-8.8) | 0.56 |
| Interleukin 8, pg/ml | 8.4 (4.7-13.1) | 8.7 (5.7-14.4) | 7.6 (4.5-12.1) | 0.30 |
| TNF, pg/ml | 8.6 (4.5-13.8) | 8.6 (5.4-14.6) | 7.7 (4.0-13.0) | 0.62 |
| Fibrinogen, g/l | 2.3 (1.7-3.4) | 2.4 (1.9-3.6) | 2.0 (1.6-2.7) | 0.07 |
| Troponin, ng/l | 4.9 (3.1-8.1) | 5.0 (3.2-8.5) | 4.8 (3.0-7.9) | 0.85 |
| Creatinine, µmol/l | 82.2 (70.7-97.2) | 81.3 (70.7-93.3) | 82.7 (70.7-106.1) | 0.57 |
| **Lung function** | | | | |
| FEV1 predicted, % | 61±19 | 58±18 | 64±20 | 0.14 |
| FVC predicted, % | 78±17 | 76±18 | 79±16 | 0.41 |
| FEV1/FVC | 60±13 | 58±12 | 61±14 | 0.22 |
| sRaw_eff_, kPa s | 1.25 (0.92-2.06) | 1.47 (1.00-2.26) | 1.11 (0.89-1.71) | 0.10 |
| ITGV, l | 4.3 (3.3-5.2) | 4.6 (3.7-5.5) | 3.9 (3.2-5.1) | 0.09 |
| TLC, l | 7.1±1.5 | 7.2±1.5 | 7.0±1.4 | 0.56 |
| RV, l | 3.3 (2.9-4.3) | 3.6 (3.0-4.3) | 3.2 (2.7-4.3) | 0.14 |
| RV/TLC, % | 51±10 | 53±11 | 49±9 | 0.08 |
| TLCO, % | 60±18 | 57±18 | 65±17 | **0.04** |
| **Echocardiographic findings** | | | | |
| LVEDD, mm | 50±7 | 51±7 | 49±6 | 0.10 |
| LVEF, % | 62 (55-70) | 60 (53-67) | 65 (60-70) | 0.06 |
| Relevant (at least moderate) mitral valve regurgitation (N=75), N (%) | 30 (40.0) | 17 (40.5) | 13 (39.4) | 1.0 |
| Left atrial diameter, mm | 41±7 | 42±7 | 40±6 | 0.21 |
| E, m/s | 0.69 (0.54-0.84) | 0.70 (0.58-0.94) | 0.68 (0.53-0.80) | 0.39 |
| A, m/s | 0.77 (0.65-0.88) | 0.76 (0.65-0.88) | 0.80 (0.63-0.88) | 0.93 |
| E/A | 0.86 (0.70-1.03) | 0.87 (0.66-1.25) | 0.85 (0.71-0.97) | 0.92 |
| Septal e´, cm/s | 7.0 (5.7-8.2) | 6.9 (6.0-8.0) | 7.0 (5.2-8.9) | 0.95 |
| E/e´ | 9.6 (7.9-12.8) | 10.0 (7.7-13.4) | 8.7 (7.9-11.8) | 0.62 |
| E(dt), ms | 227 (194-278) | 227 (194-267) | 240 (192-295) | 0.41 |
| Right ventricular wall thickness, mm | 5.6 (4.3-6.9) | 5.8 (4.1-7.0) | 5.3 (4.3-6.1) | 0.52 |
| TAPSE, mm | 24±5 | 25±5 | 24±5 | 0.52 |

6MWD, 6-min walk distance; BMI, body mass index; BODE index composed of BMI, airflow obstruction, dyspnea and 6MWD; COPD, chronic obstructive pulmonary disease; CRP, C-reactive protein; E(dt) E wave deceleration time; EQ-5D, euro quality of life l 5 dimensions questionnaire; FEV1, forced expiratory volume in one second; FVC, forced vital capacity; GOLD, global initiative for chronic obstructive lung disease; ICS, inhaled corticosteroids; IPAQ, international physical activity questionnaire; ITGV, intrathoracic gas volume; LABA, long-acting beta2-agonist; LAMA, long-acting muscarinic antagonist; LDL, low density lipoprotein; LVEDD, left ventricular end-diastolic diameter; LVEF, left ventricular ejection fraction; mMRC, modified medical research council; RV, residual volume; sRaw_eff_, effective specific airway resistance; TAPSE, tricuspid annular plane systolic excursion; TIA, transient ischemic attack; TLC, total lung capacity; TLCO, transfer factor of the lung for carbon monoxide; TNF, tumor necrosis factor

e-Table 3. Clinical and functional characteristics at follow-up of the subgroup of patients without oral anticoagulation therapy.

|  | Total Cohort (N=174) | Study group (N=58) | Control group (N=116) | *P* value |
| --- | --- | --- | --- | --- |
| **Clinical Outcome** | | | | |
| MACCE, N (%) | 23 (13.2) | 16 (27.6) | 7 (6.0) | **<0.001** |
| New-onset coronary artery disease, N (%) | 7 (4.0) | 5 (8.6) | 2 (1.7) | **0.04** |
| New-onset myocardial infarction, N (%) | 3 (1.7) | 2 (3.4) | 1 (0.9) | 0.26 |
| New-onset heart failure (N=139), N (%) | 13 (9.4) | 9 (20.0) | 4 (4.3) | **0.005** |
| New-onset stroke/TIA, N (%) | 3 (1.7) | 2 (3.4) | 1 (0.9) | 0.26 |
| **Therapy** |  |  |  |  |
| Anticoagulation therapy, N (%) | 20 (11.5) | 15 (25.9) | 5 (4.3) | **<0.001** |
| Antiplatelet therapy, N (%) | 73 (42.0) | 22 (37.9) | 51 (44.0) | 0.52 |
| Betablocker therapy, N (%) | 53 (30.5) | 26 (44.8) | 27 (23.3) | **0.005** |
| Digitalis therapy, N (%) | 3 (1.7) | 2 (3.4) | 1 (0.9) | 0.26 |
| Antiarrhythmic drug therapy:   - Class III (Amiodarone, Dronedarone), N (%) | 1 (0.6) | 1 (1.7) | 0 (0) | 0.33 |
| Respiratory medication:   - LABA, N (%) - LAMA, N (%) - ICS, N (%) - LABA/LAMA, N (%) - LABA/ICS, N (%) - LABA/LAMA/ICS, N (%) | 127 (73.0)  123 (70.7)  96 (55.2)  102 (58.0)  96 (55.2)  74 (42.5) | 47 (81.0)  42 (72.4)  35 (60.3)  36 (62.1)  34 (58.6)  28 (48.3) | 80 (69.0)  81 (69.8)  61 (52.6)  65 (56.0)  62 (53.4)  46 (39.7) | 0.11  0.86  0.42  0.52  0.63  0.33 |
| Long-term oxygen therapy, N (%) | 35 (20.1) | 12 (20.7) | 23 (19.8) | 1.0 |
| **Clinical status** | | | | |
| Hypoxemia (N=173), N (%) | 80 (46.2) | 21 (36.8) | 59 (50.9) | 0.11 |
| Hypercapnia (N=173), N (%) | 6 (3.5) | 3 (5.3) | 3 (2.6) | 0.40 |
| Exacerbation within the last 12 months:   - Max. 1 and without hospital admission, N (%) - At least 2 or with hospital admission, N (%) | 106 (60.9)  68 (39.1) | 31 (53.4)  27 (46.6) | 75 (64.7)  41 (35.3) | 0.19 |
| Hospitalization since last visit, N (%) | 68 (39.1) | 27 (46.6) | 41 (35.3) | 0.19 |
| Health status since last visit:   - Improved, N (%) - Idem, N (%) - Worse, N (%) | 19 (10.9)  83 (47.7)  72 (41.4) | 3 (5.2)  26 (44.8)  29 (50.0) | 16 (13.8)  57 (49.1)  43 (37.1) | 0.11 |
| mMRC dyspnea scale ≥2, N (%) | 83 (47.7) | 31 (53.4) | 52 (44.8) | 0.34 |
| BODE index >4 (N=148), N (%) | 17 (11.5) | 9 (19.1) | 8 (7.9) | 0.06 |
| DemTect score | 15 (13-17) | 16 (14-18) | 15 (13-17) | 0.24 |
| EQ-5D utility score | 0.89 (0.79-1.00) | 0.89 (0.79-0.90) | 0.89 (0.79-1.00) | 0.33 |
| 6MWD, m | 414 (360-476) | 388 (313-470) | 425 (370-478) | 0.08 |
| IPAQ score | 2,628 (705-7,103) | 1,707 (258-4,995) | 2,795 (1,188-8,435) | **0.02** |
| Timed up&go test, s | 7.4 (6.0-9.0) | 7.5 (6.1-9.3) | 7.3 (6.0-8.5) | 0.39 |
| **Echocardiographic findings** | | | | |
| LVEDD, mm | 50±7 | 49±8 | 50±7 | 0.32 |
| LVEF, % | 60 (55-67) | 58 (50-65) | 65 (59-70) | **0.003** |
| Relevant (at least moderate) mitral valve regurgitation (N=90), N (%) | 51 (56.7) | 22 (64.7) | 29 (51.8) | 0.28 |
| Left atrial diameter, mm | 39±6 | 41±6 | 37±5 | **0.003** |
| E, m/s | 0.66 (0.57-0.80) | 0.65 (0.50-0.80) | 0.67 (0.57-0.79) | 0.77 |
| A, m/s | 0.80 (0.65-0.93) | 0.70 (0.51-0.83) | 0.82 (0.69-0.96) | **0.01** |
| E/A | 0.82 (0.70-1.02) | 1.01 (0.74-1.19) | 0.80 (0.69-0.97) | 0.08 |
| Septal e´, cm/s | 7.0 (5.9-8.0) | 7.2 (6.0-10.0) | 6.7 (5.1-7.4) | 0.05 |
| E/e´ | 10.2 (7.6-13.0) | 9.7 (7.0-11.4) | 10.3 (8.1-14.3) | 0.23 |
| E(dt), ms | 215 (174-278) | 187 (135-224) | 243 (184-310) | **0.001** |
| Right ventricular wall thickness, mm | 5.0 (4.0-7.0) | 5.4 (4.1-7.3) | 5.0 (4.0-7.0) | 0.90 |
| TAPSE, mm | 23±4 | 21±3 | 24±4 | **<0.001** |

6MWD, 6-min walk distance; BODE index composed of BMI, airflow obstruction, dyspnea and 6MWD; E (dt) E wave deceleration time; EQ-5D, euro quality of life l 5 dimensions questionnaire; ICS, inhaled corticosteroids; IPAQ, international physical activity questionnaire; LABA, long-acting beta2-agonist; LAMA, long-acting muscarinic antagonist; LVEDD, left ventricular end-diastolic diameter; LVEF, left ventricular ejection fraction; MACCE, major adverse cardiac and cerebrovascular events ; mMRC, modified medical research council; TAPSE, tricuspid annular plane systolic excursion; TIA, transient ischemic attack

e-Table 4. Uni- and multivariate regression analyses for markedly prolonged APWD of the subgroup of patients without oral anticoagulation therapy.

|  | **Univariate regression analysis** | | **Multivariate regression analysis** | |
| --- | --- | --- | --- | --- |
|  | Odds ratio (95% CI) | *P* value | Odds ratio (95% CI) | *P* value |
| Age, years | 1.04 (1.00-1.08) | **0.043** | 1.05 (1.01-1.10) | **0.018** |
| Male | 0.47 (0.20-1.12) | 0.088 |  |  |
| BMI, kg/m^2^ | 1.13 (1.05-1.20) | **<0.001** | 1.12 (1.04-1.21) | **0.003** |
| Arterial Hypertension | 1.91 (0.99-3.67) | 0.054 |  |  |
| Diabetes mellitus | 2.04 (0.91-4.55) | 0.083 |  |  |
| Hyperlipidemia | 1.68 (0.91-3.13) | 0.100 |  |  |
| Obstructive sleep apnea | 2.23 (1.04-4.76) | **0.039** | 1.45 (0.60-3.51) | 0.413 |
| Hypoxemia | 1.12 (0.60-2.08) | 0.731 |  |  |
| Exacerbation within the last 12 months | 1.02 (0.55-1.90) | 0.941 |  |  |
| Leukocytes, 10^9^/l | 1.01 (0.88-1.15) | 0.927 |  |  |
| CRP, nmol/l | 1.00 (1.00-1.00) | 0.094 |  |  |
| Alpha-1 antitrypsin, µmol, l | 1.01 (0.96-1.07) | 0.687 |  |  |
| Interleukin 6, pg/ml | 1.00 (0.98-1.02) | 0.945 |  |  |
| Interleukin 8, pg/ml | 1.01 (0.96-1.06) | 0.720 |  |  |
| TNF, pg/ml | 1.00 (0.98-1.02) | 0.754 |  |  |
| Fibrinogen, g/l | 0.95 (0.72-1.25) | 0.718 |  |  |
| FEV1, predicted % | 1.01 (0.99-1.02) | 0.494 |  |  |
| FEV1/FVC | 1.01 (0.99-1.04) | 0.231 |  |  |
| ITGV, l | 0.85 (0.67-1.06) | 0.146 |  |  |
| RV/TLC, % | 0.98 (0.96-1.01) | 0.272 |  |  |
| TLCO, % | 1.00 (0.99-1.02) | 0.481 |  |  |
| Right ventricular wall thickness, mm | 1.04 (0.93-1.17) | 0.491 |  |  |

BMI, body mass index; CRP, C-reactive protein; FEV1, forced expiratory volume in one second; FVC, forced vital capacity; ITGV, intrathoracic gas volume; RV, residual volume; TLC, total lung capacity; TLCO, transfer factor of the lung for carbon monoxide; TNF, tumor necrosis factor
